# Desire for Success Awakens: Proof of Competence Restoration in a Non-competitive Environment

**DOI:** 10.3389/fnins.2021.698777

**Published:** 2021-06-21

**Authors:** Liang Meng, Guanxiong Pei, Yupei Zhang, Jia Jin

**Affiliations:** ^1^School of Business and Management, Shanghai International Studies University, Shanghai, China; ^2^Research Center for Advanced AI Theory, Zhejiang Lab, Hangzhou, China

**Keywords:** competition, competence frustration, competence restoration, event-related potentials, feedback-related negativity, motivation

## Abstract

Pioneering studies reported that individuals who worked on a highly difficult task and experienced competence frustration beforehand would activate a restorative process and show enhanced autonomous motivation in a subsequent irrelevant activity. In this follow-up study, we explored the effect of prior competition outcome on one’s autonomous motivation in a subsequent non-competitive environment. According to our experimental manipulation, participants were randomly assigned to two treatment groups (a winning group and a losing group) and a control group. The experiment lasted for three sessions. Participants in the control group completed a single-player stop-watch (SW) task all along, while those in both treatment groups worked on a competitive SW task and competed for monetary rewards during Session 2 only. Electrophysiological data in Session 1 serve as the baseline and measure one’s trait-level autonomous motivation towards the SW game. For participants in the losing group, more pronounced difference wave of feedback-related negativity was observed in Session 3 compared with Session 1, suggesting enhanced autonomous motivation in Session 3. Such a pattern was observed in neither the winning group nor the control group. These results suggested that failure in a prior competition would activate one’s competence restoration in a subsequent non-competitive environment. Task difficulty and social competition are varied sources of competence frustration. Thus, our findings advanced understanding of the competence restorative process and helped clarify the dynamics between competition and human motivation.

## Introduction

“Whatever does not kill us makes us stronger.”

(Friedrich Nietzsche quotes)

Life without competition is life without progress. Indeed, competition is ubiquitous throughout our lives and is essential for our survival. Through competition, human beings endeavor to achieve and maintain a higher status in a social hierarchy so as to acquire scarce resources that are valuable to them (Vermeer et al., 2016). If used in a proper manner, competition can be a powerful motivator, which can effectively strengthen both autonomous and controlled motivation. While autonomous motivation refers to one’s natural tendency to be curious and interested, to look for challenges and develop skills and knowledge without external incentives (Hagger and Chatzisarantis, 2016; Meng and Ouyang, 2020), controlled motivation describes one’s engagement in an activity with the aim of attaining a reward or avoiding a punishment (Ryan and Deci, 2017). In sports tournaments, athletes are highly motivated to outperform others and to win a prize, in which case the athletes are externally driven. On the other hand, they are prompted by the internal drive of self-improvement as well as competence and skill enhancement (Vermeer et al., 2016). Indeed, competition has been found to effectively enhance human motivation and performance across varied domains, from leisure, education, the workplace, market economies to politics (Hirshleifer, 1978; Deci et al., 1981).

With the aim of clarifying effects of varied social, environmental and contextual factors on human motivation, Deci and Ryan (2000) proposed self-determination theory (SDT) from a cognitive perspective. After continuous development and refinement in the past few decades, it has formed a systematical theoretical system and become one of the most influential theories of human motivation. One major contribution of SDT is that it conceptualizes the three basic psychological needs of autonomy, competence and relatedness as innate and overarching for one’s psychological growth and well-being (Ryan and Deci, 2017). Autonomy describes the need to feel autonomous and self-directed and to identify with one’s own behaviors, while relatedness refers to the need to interpersonally and/or socially connect with others, to give love and affection, and to receive them in return. Finally, competence reflects the need to feel effective and mastery, and to demonstrate and improve one’s capabilities (Ryan and Deci, 2000, 2017). A recent meta-analysis revealed that satisfaction of the three basic psychological needs is effective in predicting one’s autonomous motivation (Van den Broeck et al., 2016).

Among the three basic psychological needs, competence is closely associated with competition. While winning a competition generally brings about competence satisfaction and is predicted to facilitate human motivation, losing a competition is often accompanied with feelings of a failure or inadequacy and doubt over one’s own ability, which are all representations of competence frustration (Bartholomew et al., 2011, 2014; Chen et al., 2015; Costa et al., 2015; Fang et al., 2017, 2018, 2019). Considerable empirical evidence has demonstrated the adverse effect of competence frustration, which substantially impairs vitality, reduced motivation and arouses defensive behaviors among adult populations (Gunnell et al., 2013; Vansteenkiste and Ryan, 2013). Similar effects have been observed in children as well. For instance, elementary school students who perceived the in-class environment as controlling experienced competence frustration and reported lower levels of subjective vitality and engagement (Earl et al., 2017). At first glance, converging evidences would suggest the detrimental effect of failing at a competition. However, it should be noted that all the existing findings revealing the “dark side” of competence frustration examined its instantaneous effect. In comparison, the intertemporal effect of competence frustration, which might be counter-intuitively motivating, was neglected in most studies.

Distinguished from other existing studies, Sheldon and Gunz (2009) empirically demonstrated that psychological need frustration could awaken one’s desire to recover the missing experience. Following this line of study, Radel et al. (2014) found that students who experienced autonomy frustration beforehand had greater motivation in the next curriculum if they could regain autonomy satisfaction in it. As a restorative mechanism comparable and parallel to that of autonomy frustration, competence-frustrated ones were also predicted to perform compensatory behaviors in subsequent activities (Sheldon and Gunz, 2009). However, empirical findings in support of this hypothesis began to be published only since the last few years (Ryan et al., 2019). In a pioneering field study conducted in a university, researchers found an intriguing positive effect of competence frustration outside of its primary thwarting context, as competence-frustrated students showed enhanced autonomous motivation in learning the next less-demanding course (Fang et al., 2017). In a follow-up laboratory experiment following a strict experimental design, this finding got replicated. Specifically, the competence-frustrated participants (who were assigned an overwhelmingly difficult task beforehand) were found to have greater autonomous motivation to win in the comparatively easy task that follows, which provides them the opportunity to restore their competence (Fang et al., 2018).

Considering that losing a competition is another source of competence frustration, in the current study we would like to examine the effect of prior competition outcome on one’s autonomous motivation to win in a subsequent non-competitive environment. To be specific, we aim to explore whether successes and failures in a prior competition would influence one’s autonomous motivation after the competitive environment disappears. In other words, in case of a defeat in a competition, would an individual be trapped in lasting low mood or actively seek for a new balance through remedial/compensatory behaviors? In this study, we adopted the StopWatch (SW) task, a game which requires the participants to stop the automatically running watch within a certain time interval. The more accurate, the better (Murayama et al., 2010; Ma et al., 2014; Fang et al., 2018, 2019). Participants were randomly assigned into two treatment groups (the winning group and the losing group) and a control group. While participants in the control group were instructed to work on the solo mode SW game during all three sessions, those in the two treatment groups worked on a two-player online SW game during Session 2 instead. Since there might be individual differences in the trait-level motivation toward the SW task, for participants in the two treatment groups, we compared their autonomous motivation to win after and before competition took place. Then, we compared this discrepancy (the autonomous motivation to win in Session 1 subtracted by that in Session 3) with that of the control group. Electrophysiological data from all participants were recorded and analyzed. Specifically, we resorted to the difference wave of Feedback-related Negativity (FRN), a representative event-related potentials (ERP) component observed during feedback processing and outcome evaluation to measure one’s autonomous motivation (Ma et al., 2014; Meng and Ma, 2015; Fang et al., 2018; Shen et al., 2021).

Feedback-related negativity generally peaks between 250 and 350 ms after feedback onset and is most pronounced over the frontal-central electrodes (San Martín, 2012). It is considered as a negative deflection which looms larger for losses than gains (Hajcak et al., 2006; San Martín, 2012). In order to explain the cognitive meaning of FRN, scholars developed two mainstream theories complementary to each other, which are reinforcement learning theory and motivational significance theory. Reinforcement learning theory suggested that FRN is sensitive to the valence of outcome feedback, being more pronounced for negative feedback than for the positive one. Motivational significance theory complements reinforcement learning theory by arguing that the difference wave of FRN (d-FRN, the FRN in the winning condition subtracted by that in the losing condition) represents subjective evaluation of the motivational significance of outcomes (Gehring and Willoughby, 2002; Yeung et al., 2005; Masaki et al., 2006). If outcomes in a given experimental condition are perceived to be more motivationally significant, a more pronounced d-FRN would be observed upon feedback (Yeung et al., 2005; Fukushima and Hiraki, 2009; San Martín, 2012; Meng and Ma, 2015).

In recent years, researchers resorted to the d-FRN to measure one’s intrinsic or autonomous motivation when external incentives are absent (Ma et al., 2014; Meng and Ma, 2015; Fang et al., 2018, 2019, 2020; Shen et al., 2021). This practice is in accordance with the motivational significance account of FRN, which gets additional support from neurobiological findings. When an individual is autonomously engaged in an activity, the dopaminergic value system would respond to feedback information (Di Domenico and Ryan, 2017; Reeve and Lee, 2019). Accordingly, pioneering functional Magnetic Resonance Imaging (fMRI) studies consistently reported activation of the anterior striatum (a likely origin of FRN) during feedback evaluation, which mirrors one’s autonomous motivation level in effort-requiring tasks (Murayama et al., 2010; Albrecht et al., 2014; Strombach et al., 2015; Swanson and Tricomi, 2015). Following this line of neuroscience studies, in this study we adopted the d-FRN to measure one’s autonomous motivation.

When a person tries something new, he/she would be curious and interested, and the autonomous motivation in this task is high. As time goes on, one becomes more and more familiar with this task, and the autonomous motivation tends to decline. This phenomenon is frequently reported in previous studies (Murayama et al., 2010; Ma et al., 2014). In this study, the participants in the control group worked on solo mode SW game during all three sessions. Thus, we predicted their d-FRN to be less pronounced in Session 3 compared with Session 1. For the participants in the winning group, as their perceived competence already got satisfied during the competition in Session 2, we predicted to observe a d-FRN pattern similar with that in the control group. For the participants in the losing group, as we hypothesized that individuals who experienced competence frustration beforehand may actively seek to restore their perceived competence afterward, we predicted that they would have a more sustained autonomous motivation to win in Session 3, resulting in a significantly more pronounced d-FRN upon feedback.

## Materials and Methods

### Participants

Seventy-eight healthy, right-handed participants took part in this study (including 39 males and 39 females). They were between 17 and 26 years old, with an average age of 21.29 and a standard deviation of 2.23. All participants were undergraduate or graduate students from Ningbo University, who had normal or corrected-to-normal vision and reported no history of neurological or mental disorders. They were randomly assigned into two treatment groups (the winning group and the losing group) and a control group (26 participants in each group). It should be pointed out that, for each participant in the two treatment groups, a same-sex experimenter unknown to him/her played as the pseudo subject. This study was conducted with the permission of the internal review board of Academy of Neuroeconomics and Neuromanagement at Ningbo University.

### Procedures

**FIGURE 1:**
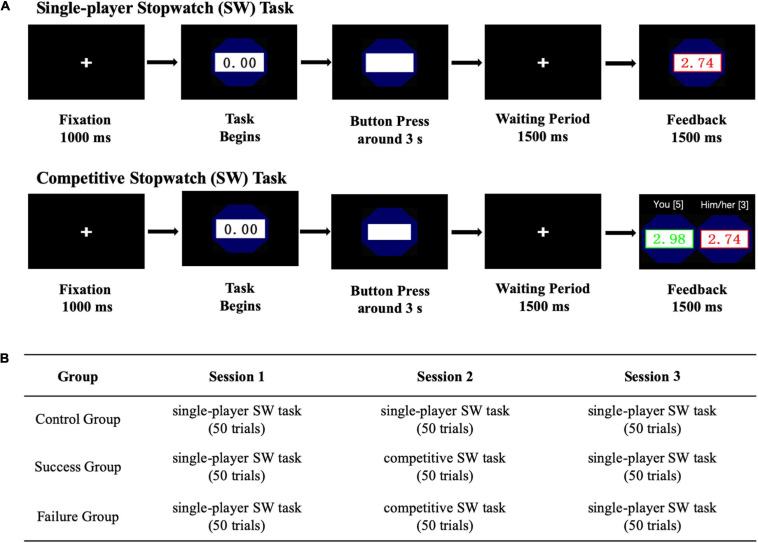
Demonstration of the experimental paradigm. **(A)** Procedures of single-player and competitive stopwatch (SW) tasks. **(B)** The experimental design and procedure.

During the experiment, the participants were comfortably seated in a dimly lit, sound-attenuated and electrically shielded experimental cubicle. Experimental stimuli were presented at the center of a computer monitor 100 cm away from the participant, with a visual angle of 6.2° × 5.4°. The experiment consisted of 3 sessions, each containing 50 trials (Figure 1B). While participants in the control group worked on a solo mode SW game adapted from Murayama et al.’s (2010) paradigm for all three sessions, those in the treatment groups were instructed to work on a two-player online SW game originally developed by Meng et al. (2016) during Session 2 only. Competition outcome was manipulated by the pseudo subject according to pre-assignment of real participants into the two treatment groups. Specially, participants who were assigned to the winning group was manipulated to win, and vice versa.

The participants were instructed to use a keypad to play the SW game all along. At the beginning of the game, a stopwatch would automatically start, and the participants should try their best to stop the watch around 3 s (Murayama et al., 2010; Albrecht et al., 2014; Ma et al., 2014). The closer, the better. The success interval was predetermined as [2.93 s, 3.07 s], which suggested medium difficulty and ensured that typical participants would succeed in about half trials according to a few previous studies (Meng et al., 2016; Ma et al., 2017; Fang et al., 2018). When playing the solo mode SW game, if the behavioral response fell within the success interval, it would be identified as a successful attempt. By contrast, the criteria of success in the two-player SW game are more complicated and rigorous. In addition to stopping the watch within the interval, one has to be more precise than his/her counterpart in order to win any single trial. The two-player SW game was competitive in nature. Points were accumulated and participant who got more points than his/her counterpart by the end of the game finally won.

As demonstrated in Figure 1A, each trial commenced with a cross symbol that lasted for 1,000 ms, and then the stopwatch would start running. During the first 2,000 ms, the participants could observe the time point on the watch in real time. Afterward, the time point would disappear, and the participants have to play the game without a cue. If the participants considered that it was time to stop the watch, they should immediately press any button they like on the keypad. Following the button press, “Button Pressed” would be displayed on the stopwatch for 1,500 ms, which was followed by the feedback. During feedback, task performances and the accumulated point were presented. In the solo mode, participants were only informed their own performances, while they were provided performances of their counterparts when playing the two-player SW game. In both modes, task performances would be displayed in a green font and with a green border for wins, which would be displayed in red instead when losing the game. It is worth pointing out that, once both responses from the paired participants fell into the success interval and were equally close to the target point of 3 s, it would be deemed as a draw. In this case, nobody would get a point and task performances would be displayed in black. There was a randomized blank interval that lasted for 600–1,000 ms.

Before the experiment formally got started, informed consents were obtained from all participants. Treatment groups’ participants met with the same-sex pseudo subject and then parted. Afterward, all participants were instructed to read the general description of the experiment. To prevent any confounds, participants were only told that the whole experiment would be divided by three sessions, and they were introduced the specific task right before the corresponding session began. To be specific, there will be a 3–5 min between-session break, during which time the participants would take a rest and learn about the following session. To familiarize them with the experimental tasks, a practice session adopting the formal task was implemented before the start of each session.

In the instruction for the solo mode SW game, participants were told that they would receive ¥10 as compensation for participation in this session. Thus, task performances were irrelevant to the payment of the session. In contrast, the winner of the two-player SW game (who accumulated more points by the end of the game) would get ¥20 as reimbursement for the session, while the counterpart would get nothing in return. The two experimenters who played as pseudo subjects had adequate training and practice before the experiment and were experts of the experimental game, which made it possible for them to manipulate the competition outcomes of the two treatment groups during Session 2. Participants who were manipulated to win fell into the winning group, and vice versa. After the experiment, subjects in the treatment groups were instructed to complete a survey on their perceived competence frustration during Session 2, after which they were debriefed and paid. The scale was adapted by Fang et al. (2019) for usage in psychology experiments, which originated from the “Work Domain Basic Psychological Need Satisfaction and Frustration Scale” (Chen et al., 2015). The sample item of the adapted scale is “I have serious doubts about whether I can play the stop-watch game well.” It is a pity that, we neglected to collect this self-rating from the first eight participants of each treatment group. Thus, only 18 participants from each treatment group responded to the post-experiment survey. Experimental stimuli, recording triggers, and response data were presented and recorded by E-Prime 2.0 (Psychology Software Tools, Pittsburgh, PA, United States).

### Measures

The EEG data were recorded with Neuroscan Synamp2 amplifier, using an EEG cap with 64 Ag/AgCl electrodes mounted according to the extended international 10–20 system. Channel data were online band-pass-filtered from 0.1 to 100 Hz and recorded at a sampling rate of 500 Hz. The left mastoid served as the on-line reference, and the EEG was off-line re-referenced to the mathematically averaged mastoids. Impedances were kept below 10 kΩ throughout the experiment. During off-line data analyses, EEG data were pre-processed adopting Curry 7.0 (Compumedics Neuroscan, Australia). Ocular artifacts were identified and corrected with the eye movement correction algorithm proposed by Miller et al. (1988), and the EEGs went through a digital low-pass filter at 30 Hz (24 dB/octave). For the FRN, epoch measurement began 200 ms prior to and ended 800 ms after onset of the feedback, with the activity from −200 to 0 ms serving as the baseline. For each participant, the recorded EEGs over each recording site were averaged across each experimental condition. Trials containing amplifier clipping, bursts of electromyography activity, or peak-to-peak deflection that exceeded ±80 μV were excluded from the final averaging procedure.

## Results

The aim of this study is to examine one’s competence restoration in a non-competitive environment. Accordingly, our experimental manipulation was implemented in Session 2, while what we are really interested in is whether the discrepancy of the amplitude of the d-FRN (the FRN in the winning condition subtracted by that in the losing condition, which measures one’s autonomous motivation to win) between Session 3 and Session 1 (data in Session 1 serve as the baseline and measure one’s trait-level autonomous motivation in playing the SW game) would be more pronounced in the losing group compared with other groups. Thus, data in Session 2 were not analyzed. Based on grand averaged waveforms of the FRN and its anterior distribution reported in previous literatures (Zhou et al., 2010; San Martín, 2012; Meng and Ma, 2015; Fang et al., 2018; Wei et al., 2020) as well as observed in this study, amplitudes from the electrodes of F1, Fz, F2, FC1, FCz, and FC2 went into statistical analyses. Because the most negative peak of the d-FRN appeared approximately 295 ms after feedback onset, we used the mean amplitudes during the 260–330 ms time period following onset of feedback in a 3 (experimental group) × 2 (experimental session) × 6 (electrode) mixed-design ANOVA, among which experimental group is a between-subject factor. In addition, a 3 (experimental group) × 2 (experimental session) mixed-design ANOVA was conducted to compare the number of successes (i.e., behavioral results). Simple effect analyses were conducted when the interaction effect was significant. The Greenhouse-Geisser correction was applied in all statistical analyses when necessary. Finally, as a manipulation check, a paired *t*-test was applied to compare self-ratings on competence frustration between the two treatment groups (the winning group and the losing group).

The paired *t*-test indicated that the losing group participants reported themselves to be competence-frustrated to a greater extent compared with those in the winning group [*M*_win_ = 2.958 (SD = 1.086), *M*_lose_ = 4.542 (SD = 0.956); *t* (17) = 4.657, *p* < 0.001], which suggested a successful manipulation. Results from the mixed-design ANOVA on the number of successes suggested a main effect of experimental session (*F*_1_, _75_ = 5.414; *p* = 0.023). The number of successes in Session 3 (*M* = 22.884) was greater than that in Session 1 (*M* = 21.154), illustrating a learning effect. The main effect of experimental group was not significant (*F*_2_, _75_ = 2.314; *p* = 0.106), nor was the interaction effect between experimental session and group (*F*_2_, _75_ = 0.493; *p* = 0.613).

**FIGURE 2:**
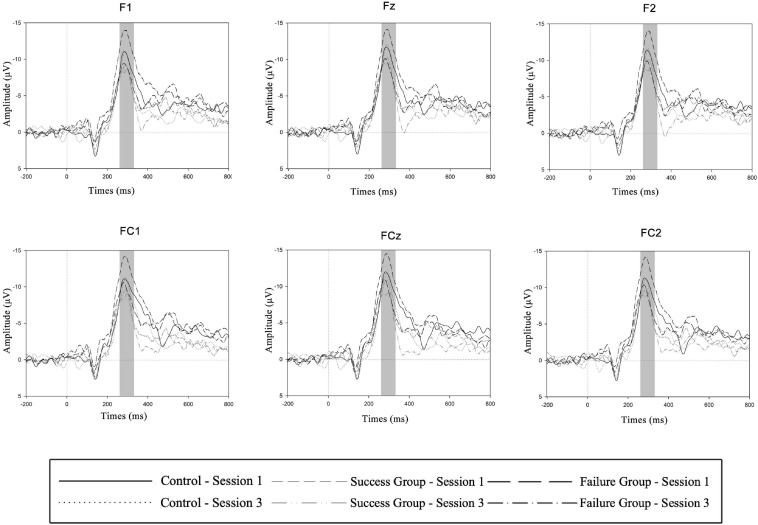
d-FRN results upon feedback. Grand-averaged d-FRN waveforms from electrodes F1, Fz, F2, FC1, FCz, and FC2 are shown for group (control group, success group and failure group) and experimental session (session 1 and session 3) conditions.

**FIGURE 3:**
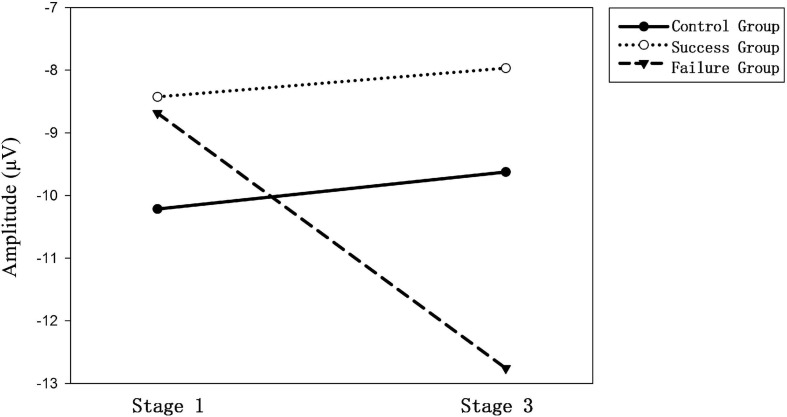
Demonstration of the interaction effect between experimental group and experimental session on the mean d-FRN magnitude.

Table 1 shows the mean d-FRN amplitude in the 3 (experimental group) × 2 (experimental session) conditions (also see Figure 2). A mixed-design ANOVA for the d-FRN revealed an interaction effect between the experimental group and the experimental session (*F*_2_, _75_ = 4.512; *p* = 0.014; see Figure 3). The main effects of both experimental group (*F*_5_, _375_ = 1.825; *p* = 0.168) and experimental session (*F*_1_, _75_ = 1.949; *p* = 0.167) were not significant. Subsequent simple effect analyses results suggested that, the d-FRN was more pronounced in Session 3 compared with Session 1 only in the losing group (*F*_1_, _51_ = 8.019; *p* = 0.007). Similar effects were not found in the winning group (*F*_1_, _51_ = 0.098; *p* = 0.755) or the control group (*F*_1_, _51_ = 0.096; *p* = 0.757).

**TABLE 1:** Mean amplitudes of the d-FRN in the 3 (experimental group) × 2 (experimental session) conditions.

Experimental group	Experimental session	Mean (μV)	Standard error	95% Confidence interval	
				
				Lower limit	Upper limit
Control group	Session 1	−10.215	1.346	−7.442	−12.988
	Session 3	−9.625	1.342	−6.861	−12.388
Winning group	Session 1	−8.427	1.126	−6.109	−10.744
	Session 3	−7.968	0.934	−6.045	−9.891
Losing group	Session 1	−8.688	0.968	−6.695	−10.681
	Session 3	−12.759	1.063	−10.569	−14.949

## Discussion

In this study, we resorted to magnitude of the d-FRN to measure one’s autonomous motivation. This approach was inspired by a pioneering fMRI study that explored the neural underpinnings of motivational processes (Murayama et al., 2010). In the two-session fMRI study conducted by Murayama et al. (2010), the well-established undermining effect of extrinsic monetary rewards on one’s intrinsic motivation was replicated. To be specific, while participants in the control group received performance-irrelevant fixed rewards during both sessions, those in the experimental group received performance contingent rewards during the first session. The blood-oxygen-level-dependent (BOLD) signal in the anterior striatum was found to prominently decrease during Session 2, a pattern that was only observed in the experimental group participants.

Given that this study is highly illuminating, it neglected to control the fundamental individual differences. People vary in their inherent intrinsic motivation toward a certain task. Considering that the typical sample size in neuroscientific investigations is rather small, the observed neural pattern might actually be driven by group-level differences in trait intrinsic motivation rather than the undermining effect. For instance, in the extreme case that participants in the control group had a greater interest in the experimental task compared with their counterparts, a similar result would be observed even if the undermining effect did not take place. As a complement to this pioneering study, in a follow-up electrophysiological study, researchers designed a three-session experiment to further examine this effect (Ma et al., 2014). While participants in the control group received performance-irrelevant fixed rewards all along, those in the experimental group received performance contingent rewards during the second session. Magnitude of the d-FRN during Session 1 served as the baseline level of one’s intrinsic motivation towards the SW task, and the difference value of d-FRN magnitude during Session 1 and Session 3 was calculated to learn whether the undermining effect indeed took place. In accordance with the above-mentioned study (Ma et al., 2014), in the current study we designed a three-session experiment to examine the effect of prior competition outcomes (i.e., successes and failures) on one’s autonomous motivation in a subsequent non-competitive environment.

Competition has existed for as long as the origin of mankind. Indeed, even the earliest human beings were involved in certain forms of competition. While it has been well established that competition has a strong influence on one’s motivation, conflicting findings were reported and few (if any) studies have examined the non-instantaneous effect of competition. This study attempts to explore the potential intertemporal effect of competition on one’s autonomous motivation. We aimed to reveal the “bright side” of competition, especially for competitors who suffered a failure. Indeed, in contrast to the widely held notion that failing at a competition would be devastating (Deci et al., 1981; Vallerand et al., 1986), it seemed that people did not passively surrender to a defeat in this study. Rather, participants in the losing group were found to have a more pronounced d-FRN (which suggested enhanced autonomous motivation to win) in the subsequent non-competitive environment. In other words, while the instantaneous losing outcome can be disappointing and detrimental, it was found to enhance one’s desire to regain the competence-fulfilling experience afterward. In contrast to the losing group, we observed declines (although non-significant declines) in autonomous motivation from Session 1 to Session 3 in participants in both the winning- and the control group, which might reflect accommodation, the natural decline in autonomous motivation as the task becomes more and more familiar to them (Ma et al., 2014).

Failing at a competition could undermine one’ perceived competence and bring about competence frustration (Vansteenkiste and Deci, 2003; Chen et al., 2015). According to findings of the subjective ratings in this study, participants in the losing group were self-reported to be competence-frustrated to a greater extent compared with those in the winning group. Competence is among the basic psychological needs proposed by SDT (Ryan and Deci, 2000, 2017). A group of researchers suggested that, only unmet or frustrated need would serve as a motivating force (Sheldon and Gunz, 2009). To be specific, recent evidences suggested that, when faced with need frustration, individuals may implement self-regulation and actively try to restore it (Veltkamp et al., 2009; Ryan and Deci, 2019). When it comes to competence frustration, in a few pioneering studies, prior competence frustration was found to initiate a restorative process, as individuals who experienced competence frustration beforehand would try to regain competence and are eager to prove themselves in another activity (Fang et al., 2018, 2019; Waterschoot et al., 2020). For instance, in an experimental study, while participants in the experimental group were asked to complete a highly difficult task during the first session and a task of medium difficulty during the second session, those in the control group were instructed to work on tasks of medium difficulty during both sessions. Similar with results of the current study, magnitude of the d-FRN was adopted to measure one’s autonomous motivation. Results suggested that participants who experienced competence frustration beforehand have enhanced autonomous motivation to win in a subsequent competence-supportive task (Fang et al., 2018).

It is worth noting that, in the vast majority of existing studies, competence satisfaction and/or frustration were manipulated through the setting of varied task difficulties (Danner et al., 1981; Locke et al., 1981; Brehm and Self, 1989; Fang et al., 2018). In the current study, a competitive environment was set up, and competence frustration was introduced as the result of failing at a competition. Task difficulty and social competition can be regarded as two different sources of competence frustration (Ryan and Deci, 2017). Thus, findings of this study supplemented and extended existing literatures on the restorative process of competence frustration. Besides, previous literatures emphasized the importance of context change during need restoration, as it was suggested to be highly difficult for one to restore the frustrated need in the primary thwarting context (Radel et al., 2014; Fang et al., 2017), which explained why participants were instructed to work on different tasks during the two sessions in the experiment conducted by Fang et al. (2018). In the current study, while participants worked on the same SW task throughout the experiment, they worked within different contexts as well. While they competed with each other during Session 2, they worked in a non-competitive environment during both Session 1 and Session 3. Correspondingly, we found that individuals would work even harder in a non-competitive environment to restore their competence undermined in prior competitions.

In a recent article, Tse et al. (2019) proposed a lifespan developmental perspective on flow theory, which incorporated the contemporary view of lifespan human development. The basic idea is that individuals would pursue and maintain flow experience across the lifespan. According to the revised imbalance-flow experience model, when faced with challenge-skill imbalances, individuals may either engage more in the affected domains and try to resolve the imbalances (continuous flow maintenance responses) or disengage from the imbalanced domains and pursue flow experience from other sources (alternative flow pursuit responses). In the pioneering study conducted by Fang et al. (2018), participants worked harder on another task of medium difficulty after experiencing competence frustration, which is an alternative flow pursuit response. In this study, participants had enhanced autonomous motivation working on the same task that brought about competence frustration to them, which can be regarded as a continuous flow maintenance response. Taken together, findings of the two studies provide additional support for the newly proposed lifespan developmental perspective on flow theory.

Findings of this study suggested that competition should be treated with the view of dialectics, which is especially true when we consider the non-instantaneous effect of competition. Indeed, failures in a competition do not always bring about detrimental consequences. Quite on the contrary, participants who experienced competence frustration due to a failure in the competition actually had a greater autonomous motivation to win in a subsequent non-competitive environment. While this finding seems to be promising, it is strongly discouraged to deliberately introduce competence frustration so as to “motivate” an individual. After all, competence restoration is a spontaneous compensatory process, which tries to compensate for one’s competence which got frustrated beforehand (Fang et al., 2018). In addition, the outcompeting status does not always maintain its edge. For one thing, one may experience a natural decline in autonomous motivation after winning a game. For another, as demonstrated by Schurr and Ritov (2016), winning a competition may engender unethical behaviors in a subsequent unrelated task, especially when the outcomes are relatively defined. Taken together, these findings suggested that the profound and lasting effects of competition can be more complicated than they seem to be. Thus, people should be really cautious when considering the setup of a competitive environment.

Given the above merits and contributions, this study is not without limitations. As has been introduced in the methods section, we neglected to collect the manipulation check data, that is the self-rating of competence frustration from the first eight participants in each treatment group. Thus, the reported significant difference in competence frustration was based on the other 36 participants that came from the winning group and the losing group. Besides, subjective ratings of one’s autonomous motivation level were not collected, which could have added additional support to the electrophysiological findings of this study.

The current study opens up new directions for future research. In follow-up studies, researchers may continue to examine the effect of competition-induced competence frustration on one’s autonomous motivation. While we observed a restorative process of competence in this study, it is worth noting that the participants only experienced a relatively short-term competence frustration (1 out of 3 sessions). Thus, whether a similar restorative response would still be observed if they experience persistent competition-induced competence frustration (e.g., a succession of failures) warrants further investigation. In addition, as a failure could be further differentiated between a near miss and a complete defeat, future researchers may control the score gap between the paired participants, such as the case in several previous studies (Meng et al., 2016; Ma et al., 2017). A near miss and a complete defeat may bring about different levels of competence frustration. As the lifespan developmental perspective on flow theory suggested that one’s response to competence frustration may vary across different situations (Tse et al., 2019), it would be illuminating to examine the effect of the level of competition-induced competence frustration on one’s subsequent restorative processes.

## Conclusion

In a three-session experimental study, we examined the effect of competition outcome on one’s autonomous motivation. During Session 1 and Session 3, participants in the two experimental groups worked on a SW task in solo mode. During Session 2, they worked on a competitive SW task and competed with each other for monetary rewards. Participants in the control group worked on the single-player SW task all along. Electrophysiological evidence suggested that, after losing the competition participants had enhanced autonomous motivation to win (as reflected in the magnitude of d-FRN in Session 1 subtracted by that in Session 3) in a non-competitive environment that follows. Such an effect was not observed in the winning group or the control group. Findings of this study advanced our understanding of the competence restorative process and helped polish the dynamics between competition and human motivation.

## Data Availability Statement

The raw data supporting the conclusions of this article will be made available by the authors, without undue reservation.

## Ethics Statement

This study was reviewed and approved by Scientific Review Committee of School of Business and Management, Shanghai International Studies University. The participants provided their written informed consent to participate in this study.

## Author Contributions

LM and GP conceived and designed the study, interpreted the data, and drafted the manuscript. GP conducted the experiment, collected the data, and analyzed the data. LM and JJ administered the project. All authors reviewed and edited the manuscript.

## Conflict of Interest

The authors declare that the research was conducted in the absence of any commercial or financial relationships that could be construed as a potential conflict of interest.
